# Trait emotional intelligence in competitive sports: are there differences in dimensions of emotional intelligence when comparing different sports?

**DOI:** 10.1186/s40359-025-02563-w

**Published:** 2025-03-15

**Authors:** Alexandra Kopp, Darko Jekauc

**Affiliations:** 1 Department of Sport Science, Humboldt University of Berlin, Berlin, Germany; 2 Department of Sport Science, Karlsruhe Institute of Technology, Karlsruhe, Germany

**Keywords:** Emotional intelligence, Trait EI, TEIQue, Type of sports, Emotional challenges, Appraisal

## Abstract

Emotional intelligence (EI) is a determinant of athletic performance. Since each sport has its own psychological requirements, individual EI dimensions could have different relevance for athletic performance. This study investigated EI variations among athletes participating in 48 different sports, considering the specific emotional challenges faced by athletes in competitive sports using the appraisal theory of emotions. A total of 605 athletes (44.3% female) actively engaged in competitive sports completed the Trait Emotional Intelligence Questionnaire via an online survey. Analyses of variance, controlling for age and gender, revealed no overall link between EI and different sports. However, the dimension of self-control within EI demonstrated significant relevance among athletes in contact sports, sports with a higher degree of control over environmental conditions, and sports with direct referee intervention. On the other hand, the dimension of well-being within EI exhibited significant relevance for athletes in individual sports. Thus, this study suggests that investigating appraisal processes in athletes’ emotional experiences, particularly focusing on the self-control dimension of EI, useful for future research in the field of EI.

**Supplementary Information:**

The online version contains supplementary material available at 10.1186/s40359-025-02563-w.

Competitive sport is a context known for its high emotional intensity, where emotions serve as signals of the significance of the situation [1–4]. The nature, timing, and intensity of these emotions can either facilitate or hinder athletic performance [2, 3, 5–9]. Consequently, athletes who possess the ability to recognize, comprehend, and effectively employ emotions to their advantage have a distinct competitive edge over those who lack such competencies [10–16]. Consequently the concept of emotional intelligence (EI) has become popular in sport psychology [17, 18].

EI, as described by Mayer and Salovey [19], encompasses inter-individual differences in the recognition, understanding, expression, regulation, and utilization of one’s own emotions and the emotions of others. The concept of EI has historically been subject to criticism and debate, particularly regarding two contrasting perspectives: the ability-based approach and the personality-based approach [19–23]. The ability model, derived from the original framework by Salovey and Mayer, conceptualizes EI as a cognitive ability closely linked to general intelligence [19]. In contrast, the trait model defines EI as a collection of emotion-related personality traits and behavioral dispositions, positioning it firmly within the domain of personality and thereby creating a clear distinction between EI as an ability and as a trait [24]. Mikolajczak’s [25] tripartite model of EI represents a comprehensive integrative approach, combining both ability-based and trait-based perspectives of EI within a unified theoretical framework by incorporating three components: knowledge, ability, and trait. This comprehensive model has been employed in the field of sport psychology to assess athletic performance and facilitate the development of EI training programs [26].

The present study focuses on the trait level of EI, which pertains to the enduring aspects of athletes’ emotional experiences [3]. Trait EI refers to the disposition of individuals in emotional situations, encompassing their typical behavioural patterns [25] and is commonly assessed through self-report questionnaires [27].

Initial research on EI in sports dates back to 2001 [17], and since then, numerous empirical studies have examined the relationship between EI and psychological aspects in sports, such as mental abilities [28, 29], motivation [30, 31], emotional experiences during competition [32, 33], unpleasant emotions such as perceived anxiety [34, 35], or stress [36]. In recent years, research on the relationship with objective physiological and hormonal parameters has also gained significance [36–39].

Research interest in the significance of EI for various sports, as well as the comparison of EI levels among athletes in different sports, remains consistently high [40–47]. To date, studies investigating EI across different sport types have yielded conflicting results. While some research suggests that athletes in contact sports or team sports exhibit higher emotional regulation and self-awareness skills [30, 48], other studies indicate no significant differences between individual and team sports or between different sport categories [40, 49–52]. These inconsistencies may stem from variations in study methodologies, the theoretical framing of EI, or differences in sample composition. Given these mixed findings, formulating precise hypotheses for each EI dimension across sport types remains challenging. Furthermore, it is important to emphasize that existing research has largely categorized sports using broad classifications, such as individual vs. team sports, or contact vs. non-contact sports. However, these classifications may overlook key psychological demands that shape athletes’ EI in competition, as the psychological demands experienced in different sports exhibit considerable variability [53–55]. Various sport disciplines differ in terms of factors such as intensity of interaction with teammates or opponents, stressors, pressure situations, strategic requirements, environmental conditions, and training approaches, among others. Therefore, when investigating the importance of EI for different sports, it is imperative to account for the psychological demands specific to each sport [56]. For these reasons, we adopt an exploratory approach to examine whether systematic patterns emerge when categorizing sports based on their emotional demands. This approach provides a foundation for refining theoretical models and guiding future hypothesis-driven research. In the subsequent section, we introduce a conceptual framework designed to address this aspect.

## Appraisals of sport situations related to the emotional demands of sport

Drawing upon appraisal theories of emotion [57], this study acknowledges that athletes engage in situational appraisals in sports, which serve as the foundation for the emergence of emotions. Our study focuses on examining the common situational appraisals in various sports using Ortony, Clore, and Collins’ (1988) general psychological emotion theory. This theory is particularly valuable as it provides a comprehensive framework for understanding the process of appraising situations and offers a precise definition of appraisals, distinguishing it from other sport-related emotion models (e.g., Hanin, 2012; Lazarus, 2000).

Ortony et al. [58] categorized three distinct classes of emotions associated with the appraisal of a (competitive) situation: reactions to events (pleased vs. displeased), reactions to agents (approving vs. disapproving), and reactions to objects (liking vs. disliking). Events can be further classified into two subcategories based on whether they pertain to oneself (prospect-based or well-being) or to others (fortunes-of-others). Well-being emotions (e.g., joy) arise in response to events that have already occurred, whereas prospect-based emotions (e.g., hope, fear) are linked to anticipated future events.

**Fig. 1:**
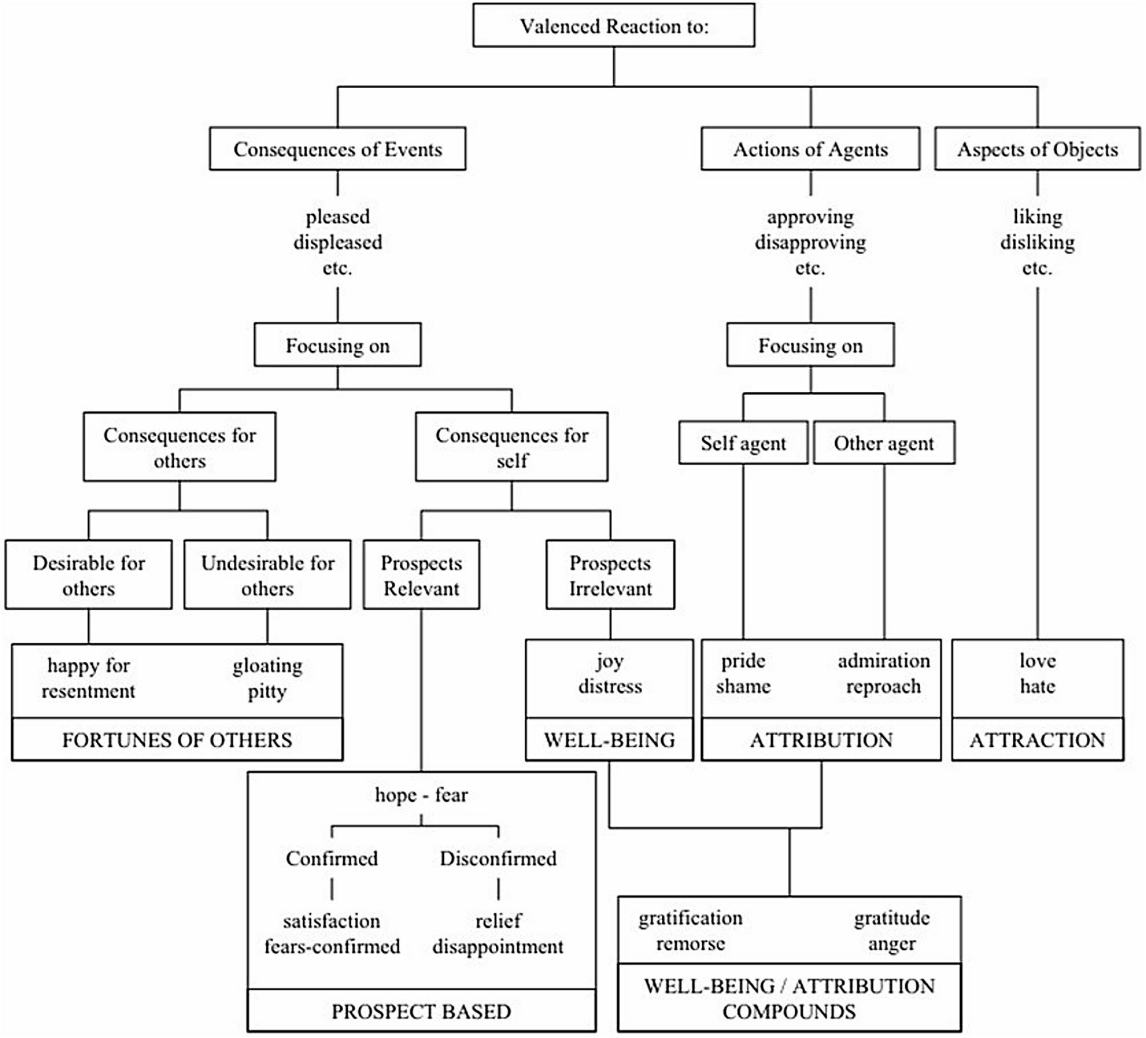
Global Structure of Emotion Types. *Note*: Adopted from Ortony, Clore, & Collins, 1988, page 19

Agents’ emotional reactions to future events can be classified into four attribution groups based on expectations. When negative expectations of an event are confirmed, the agent may experience “fear-confirmed” or “relief” depending on the outcome. Conversely, positive expectations can give rise to “satisfaction” or “disappointment.” Appraisals of one’s own actions can result in “pride” or “shame,” while evaluations of another’s actions can elicit “admiration” or “blame.” Lastly, reactions to objects can evoke feelings of “liking” or “disliking” [59]. Figure 1 provides an overview of the global structure of emotions types.

Drawing from the presented theoretical model, we have identified five key aspects that enable the categorization of sports while considering emotional demands: [1] social interaction [2], presence of an opponent [3], physical contact [4], degree of control over environmental conditions, and [5] influence of referees. In the following sections, each of these aspects will be elaborated upon in detail.

### Social interaction

**Fig. 2:**
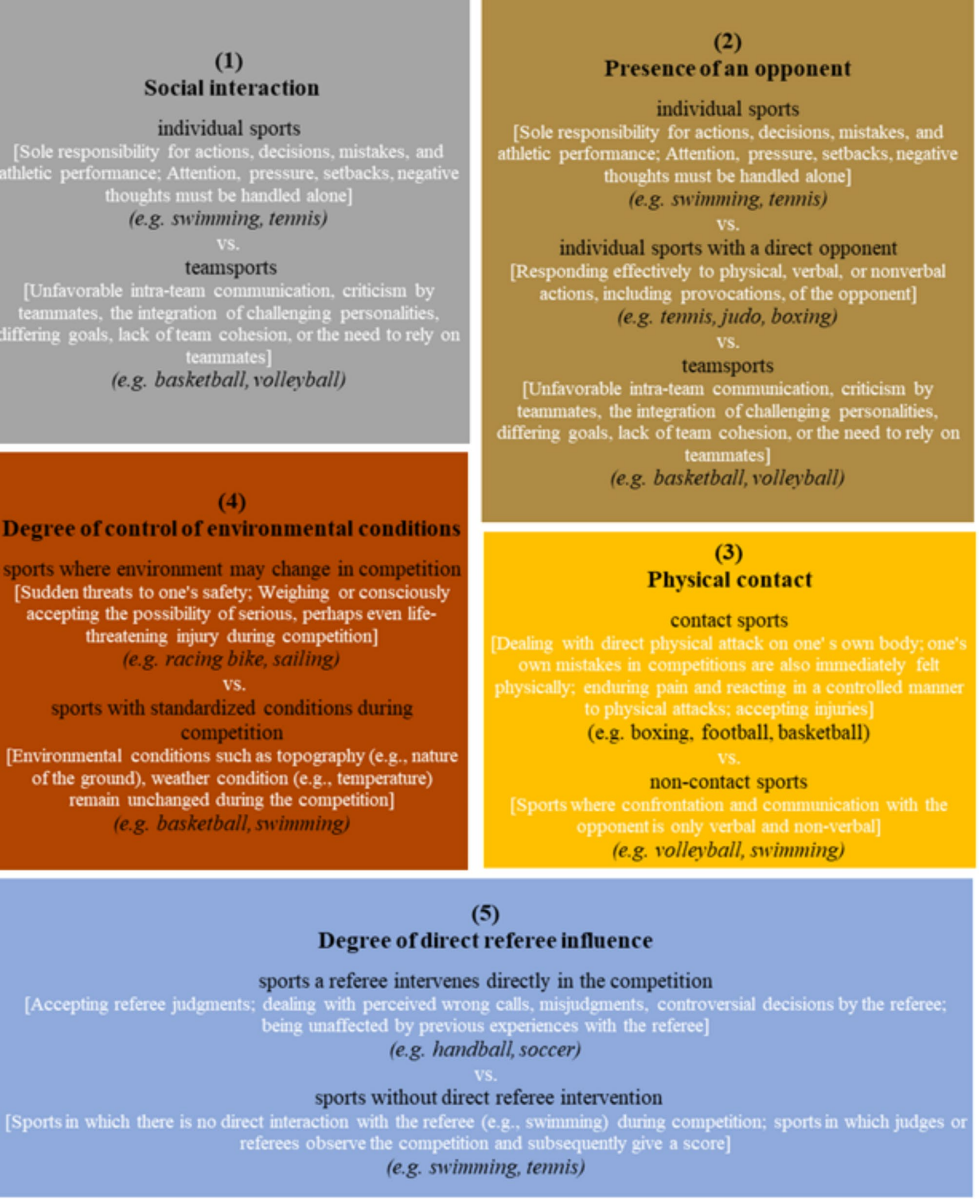
Categorization concept of types of sports

Social interaction plays a crucial role in shaping the emotional experiences of athletes within the sporting context [60, 61]. The extent of social interaction, encompassing interactions with teammates, coaches, and spectators, has been shown to have a significant impact on athletes’ emotional states. In individual sports, such as swimming or tennis, athletes bear the sole responsibility for their performance, decisions, and managing their emotions [40, 62]. They must independently process their thoughts, manage pressure, and cope with setbacks without direct support from teammates during competition. While this fosters self-reliance and autonomy, it also increases the cognitive and emotional burden on the athlete [63, 64]. Conversely, team sports, such as basketball or volleyball, involve a collective responsibility for performance, allowing athletes to share both successes and failures [61, 65–67]. This shared experience can alleviate individual performance pressure and provide opportunities for social support and motivation from teammates [68]. However, it is important to acknowledge that interacting with team members can also introduce uncertainties and emotionally demanding situations, such as intra-team communication challenges, criticism from teammates, integration of dominant personalities, or the need to rely on others [69–72]. According to Ortony’s theory of emotion, teammates significantly contribute to the appraisal of competitive situations by influencing how athletes perceive the event, the agent, and the object [58]. While individual sport athletes must internally regulate their emotions with limited external feedback, team sport athletes experience a dynamic interplay of emotions shaped by social interactions. To ensure conceptual consistency in our categorization (Fig. 2), we consider social interaction in individual sports as the degree to which athletes manage and process emotions independently, whereas in team sports, social interaction includes both supportive and challenging interpersonal dynamics that influence emotional regulation. Consequently, it is hypothesized that there may exist differences in EI between athletes engaged in individual and team sports, given the varying social dynamics and demands associated with these sports.

### Presence of an opponent

The presence of an opponent significantly influences the emotional experiences of athletes participating in individual sports and team sports involving direct competition (e.g., tennis, judo, boxing, basketball, football), as they must effectively respond to the opponent’s physical, verbal, or nonverbal actions, including provocations [26, 73–76]. Competing against an opponent introduces an external source of uncertainty and psychological pressure, requiring athletes to regulate their emotions in response to the actions and strategies of others. According to Ortony et al. [58], the presence of an opponent in a competitive situation constitutes is an additional factor in evaluating the situation. When athletes focus on the event’s consequences for the opponent, it can result in gloating over the opponent’s mistake or resentment over a successful attack. The way athletes appraise their opponent’s actions (e.g., praiseworthy, blameworthy) can influence the direction (e.g., anger, remorse) and intensity of emotions experienced during competition [59]. Moreover, the opponent can become a central object of emotional appraisal, eliciting emotions such as admiration, frustration, or hostility, depending on prior interactions and expectations [59, 77].

In contrast, athletes competing in sports without a direct opponent (e.g., running, gymnastics, golf) experience different emotional challenges. Without an opponent as an external reference point, these athletes must primarily regulate their emotions in response to internal factors, such as personal performance expectations, self-motivation, and perceived progress toward goals. The absence of an external competitor shifts the appraisal process toward self-comparison and the evaluation of personal effort rather than reactive responses to an opponent’s actions. In these sports, EI may be particularly relevant for managing self-induced pressure, maintaining focus in the absence of direct external competition, and setting realistic performance benchmarks. To ensure conceptual consistency in our categorization (Fig. 2), we define sports with an opponent as those where athletes must continuously adapt their emotions and strategies in response to an external competitor, while sports without an opponent are characterized by self-regulated emotional processes without direct interpersonal confrontation. Consequently, it is hypothesized that differences in EI may exist between athletes engaged in sports with and without a direct opponent.

### Physical contact

The role of physical contact in sports varies significantly across different disciplines, influencing both the psychological demands placed on athletes and their emotional regulation strategies. Contact sports, such as boxing and football, pose unique challenges due to the partial legitimization of aggressive actions within the rules of these sports [45, 54]. Athletes participating in these sports must develop the ability to manage their emotions in the face of physical contact, including tolerating pain without fear, accepting the risk of injury, and avoiding uncontrollable reactions to physical attacks [34, 45, 54]. The experience of physical contact with opponents and the resulting discomfort can significantly influence the appraisal of competitive situations. Focusing on the unpleasant consequences, such as pain resulting from an opponent’s body hit or aggressive defensive plays involving strong physical actions or intentional fouls, can evoke distress and anger, leading to further emotional experiences for the athlete during competition [58].

In contrast, non-contact sports, such as running or golf, present a different set of emotional challenges. The absence of direct physical interactions with opponents shifts the emotional demands toward self-regulation in response to internal and environmental factors, such as pacing, technique execution, or adapting to external conditions (e.g., weather, course difficulty). While physical endurance remains a key factor in non-contact sports, emotional regulation is often centered around sustaining concentration, coping with performance pressure, and managing fatigue without external physical interference. The lack of an external physical challenge may also alter an athlete’s competitive appraisal, making self-motivation and internal goal-setting particularly important for emotional regulation. To ensure conceptual clarity and consistency in our categorization (Fig. 2), we define contact sports as those where athletes must regulate emotions in response to physical confrontations with opponents, while non-contact sports involve emotional regulation primarily in relation to internal or environmental challenges rather than direct physical interactions. Consequently, it is hypothesized that differences in EI may exist between athletes engaged in contact sports and those participating in non-contact sports.

### Degree of control of environmental conditions

The level of control athletes have over environmental conditions, such as weather, playing surface, or equipment, can have a significant impact on their emotional responses and performance. Sports with a high degree of environmental control (e.g., gymnastics, swimming, weightlifting) take place in standardized conditions where external factors remain stable across competitions. In these sports, athletes can focus on refining their technique and optimizing performance without the unpredictability of changing environmental influences. Emotional regulation in high-control sports is often centered around internal factors, such as sustaining concentration, managing competition pressure, and maintaining consistency in execution.

In contrast, sports with a low degree of environmental control (e.g., outdoor cycling, sailing, skiing) expose athletes to variable and often uncontrollable external conditions, such as sudden weather changes, terrain shifts, or equipment instability, which can pose psychological and physical challenges [78, 79]. According to Ortony et al. [58], these changes play a crucial role in appraising the competitive environment. When athletes focus on the potential impact of these changes on themselves, such as the increased risk of slipping on a wet surface during a cycling race, it can lead to distress. Moreover, if their focus is on the future consequences of these changes, such as the possibility of rain and associated risks, it can induce fear, which can be confirmed or disconfirmed when the event occurs, ultimately resulting in approving or disapproving emotions. Scherer’s [80] component process model emphasizes the role of control in the appraisal of competition situations in sports, particularly when environmental conditions can change during a competition. Athletes engage in information processing to evaluate the relevance of events, assess the probability of consequences, and compare them to their expectations of the competitive situation. They also evaluate their coping potential to handle expected consequences.

To ensure conceptual consistency in our categorization (Fig. 2), we define high-control sports as those in which external conditions remain stable, allowing athletes to focus on performance execution without adapting to environmental variability. Conversely, low-control sports are those in which environmental unpredictability is a significant factor, requiring athletes to regulate emotions in response to external and often uncontrollable situational changes. It is hypothesized that athletes in sports with high control may differ from those in sports with low control, as a lack of control is often associated with intense negative emotions [80].

### Referee influence

The presence and role of referees in sports can significantly impact athletes’ emotional experiences, particularly in relation to their perception of fairness, competition outcomes, and the ability to regulate emotions in response to external authority figures  [81]. Sports with a high degree referee influence, such as handball and soccer, require athletes to accept referees’ decisions as an integral part of competition [82, 83]. These decisions, whether perceived as fair or unfair, shape athletes’ appraisals of competitive situations, influencing emotions such as anger or satisfaction [58]. Coping with perceived incorrect calls, avoiding emotional carryover from past decisions, and maintaining composure under the potential influence of subjective officiating are key emotional challenges in these sports [78, 79]. The referee’s actions not only regulate the competition’s structure but also serve as a psychological factor that athletes must manage throughout the game.

Conversely, sports with a low degree of referee influence (e.g., sprinting, golf, archery) involve minimal or indirect intervention from officials. In these sports, outcomes are primarily determined by objective performance criteria (e.g., fastest time, most accurate shot), and referees or officials serve mainly as rule enforcers rather than active regulators of competition flow. As a result, athletes in these sports experience fewer emotionally charged interactions with referees, and their emotional regulation is less focused on coping with external judgments and more on self-monitoring and internal performance evaluation.

According to Ortony et al. [58], the appraisal of external agents, such as referees, plays a crucial role in emotional responses to competition. In sports with strong referee influence, athletes are more likely to experience emotions related to authority appraisal, fairness perception, and decision acceptance, whereas in sports with low referee influence, emotional responses are primarily driven by self-evaluation and competition outcomes.

To ensure conceptual clarity in our categorization (Fig. 2), we define high-referee-influence sports as those in which referees actively shape the competition and make real-time decisions affecting athletes’ actions, while low-referee-influence sports are those where officials play a limited role and outcomes are primarily performance-based. Therefore, it is hypothesized that differences exist between athletes in sports with a strong referee influence and those in sports with a low referee influence. An overview of the identified categories of the sport is provided in Fig. 2.

Given the conflicting results in previous research, this study takes an exploratory approach to examine EI differences among athletes across various sport types. Rather than testing predefined hypotheses, we aim to identify systematic patterns in EI dimensions based on distinct emotional demands in competitive sports. This approach allows for a more refined understanding of EI in different sport contexts and provides a foundation for future hypothesis-driven research. The primary objective of this research is to examine the differences in EI levels among athletes across various sport groups. To classify the sport groups, we have drawn upon the theoretical framework of appraisal theory of emotions to identify distinct emotional demands encountered by specific sport groups. The central aim is to discern EI characteristics across different sports, with a focus on five aspects: social interaction, presence of an opponent, physical contact, degree of control of environmental conditions, and referee influence. The study adopts an exploratory approach to examine the hypothesized differences in the level of EI and its subcategories (i.e., well-being, self-control, emotionality, and sociability) due to the inconsistent findings reported in previous research [30, 40, 47, 48, 51, 84–86]. This study aims to enhance our understanding of the emotional experiences and demands across distinct sports by investigating disparities in the dimensions of EI levels among athletes participating in various sport types.

## Method

A quantitative, non-experimental, observational study with a cross-sectional design was employed to investigate the differences in dimensions of EI levels among athletes engaged in different sports.

### Procedure

An online survey was conducted with the survey period lasting from 1 February 2019 to 27 June 2019. The sample was obtained through quota sampling, specifically targeting athletes actively participating in competitive sports. Therefore, a total of 44 federal professional associations (Bundesfachverbände), 132 regional associations (Landesfachverbände), 16 national federation of sports (Landessportbünde), and 121 sport clubs were contacted through existing networks via email, and in some cases by telephone, to request their support for the study. This strategy aimed to leverage the communication networks of these organizations effectively, facilitating the recruitment of a large and diverse sample of athletes across multiple sports disciplines. Email content included information about the current study and a prepared call for participation in the study for distribution to the members. The survey, which took approximately 15 min to complete, was voluntary, and participants had the right to withdraw at any time without providing a reason. Data was anonymized before analysis. Ethics approval was obtained from the Ethics Committee of the Faculty of Humanities and Social Sciences at the Humboldt University in Berlin (No. HU-KSBF-EK_2019_0001).

### Participants

The study included a total of 605 (44.3% female) athletes who were active in competitive sports at the time of the survey. On average, the participants practiced their sport for 13.8 years (*SD* = 9.3), practiced four times per week (*SD* = 2.5) for approximately seven hours a week (*SD* = 6.0). Almost all athletes were German citizens (96.2%). In the current sample, a diverse range of 48 distinct sports is included.

### Instruments

In addition to the informed consent, demographic and sport-specific data, participants answered the TEIQue-SF in the German version.

#### Trait emotional intelligence questionnaire short form (TEIQue-SF)

The German version of the Trait Emotional Intelligence Questionnaire (TEIQue-SF) was used to assess Trait EI in a German-speaking sample [87]. This 30-item questionnaire utilizes a 7-point Likert scale (1 = *completely disagree*, 7 = *completely agree*). The TEIQue-SF consists of a global trait EI and four factors: *well-being* (self-esteem, trait optimism, trait happiness), *self-control* (emotion regulation, stress management, low impulsiveness), *emotionality* (emotion perception, emotion expression, trait empathy, relationships), and *sociability* (assertiveness, social awareness, emotional management) [87]. A detailed description of the individual dimensions can be found in Appendix A. Laborde et al. [88] have evaluated the validity of the TEIQue’s factor structure in the sports context. The internal consistencies of the German version of the TEIQue-SF in their validation study ranged from 0.86 to 0.94 at the factor level, with a Cronbach’s *α* of 0.96 for the global trait EI [87]. The TEIQue-SF is considered a reliable instrument for assessing EI in sports and is recommended for use in sports psychology practice [13, 52].

### Statistical data analysis

To categorize the types of sports based on their emotional demands, our analysis focused on five different group constellations: [1] Social interaction [2], Presence of an opponent [3], Physical contact [4], Degree of control over environmental conditions, and [5] Referee influence. For a more comprehensive understanding, Fig. 2 provides a detailed overview of these group constellations and the corresponding comparisons.

Based on the study result on EI in athletes of different sport categories by Castro-Sánchez et al. [30], we computed a priori power analysis with G*Power [89] for a univariate variance analysis (fixed effects, omnibus, one way), with an estimated effect size (*f* = 0.33) with a power of 0.80 and an alpha level of 0.05, which estimated a necessary sample size of *n* = 90 for three groups, and a necessary sample size of *n* = 74 for two groups. To examine the relationship between trait EI and the type of sports, we conducted five one-way ANOVAs. One-way ANOVAs were chosen over a multivariate approach to independently examine each EI dimension’s unique relationship with sport type, avoiding assumptions of interdependence that lack theoretical support in this context. The dependent variable comprised the four trait EI factors and the overall trait EI score, while the independent variable was the type of sport. Gender was included as an additional factor, and age was considered as a covariate in the analyses. For the two control variables, the results are reported only when significant effects were observed. Missing data were assessed using Little’s MCAR test, and the analysis confirmed no significant deviations. Results of the one-way ANOVAs are presented as *F*-values, *p*-values, Eta-square (*η²*), and degrees of freedom (*df*). A Bonferroni post-test was performed to identify specific differences when significant main effects were found in the comparison of three groups. Significance level was set at 5% level. All statistical analyses were performed using IBM SPSS Statistics (Version 25).

## Results

The online survey achieved a response rate of 19.1%. In total, the survey was accessed 1,659 times, with 717 participants initiating the interview. Of these, 658 participants completed the survey in full, and ultimately, 605 datasets were included in the study. Table 1 provides descriptive statistics on socio-demographic information, Table 2 presents an overview of athlete’s participation by sport type, and Table 3 shows descriptive statistics on EI levels.

**Table 1:** Descriptive statistics on Socio-demographic information

	*N*	Age		Gender		Years practicing		Training week		Training hours	
		***M***	***SD***	**F**_***(N)***_	**M**_***(N)***_	***M***	***SD***	***M***	***SD***	***M***	***SD***
**Total**	605	28.2	10.7	268	355	13.9	9.3	4.2	2.5	7.0	6.0
(1) *Social interaction*											
**Individual**	272	31.4	12.3	159	113	16.9	10.4	4.4	3.0	8.8	6.2
**Team**	333	25.7	8.4	109	222	11.5	7.5	4.0	2.2	5.7	5.6
(2) *Presence of an opponent*											
**Individual** - with opp.	69	34	13.4	24	45	17.3	13.3	3.9	3.0	7.5	6.2
**Individual -** without opp.	208	31.0	11.8	132	76	16.3	9.8	4.7	2.9	9.2	6.1
**Team**	328	25.3	8.0	112	214	11.7	7.2	4.0	2.2	5.7	5.5
(3) *Physical contact*											
**Contact**	217	26.4	9.8	101	114	13.0	8.5	3.7	2.4	6.2	4.1
**Non-contact**	388	29.3	11.0	167	221	14.4	9.7	4.4	2.6	7.5	6.9
(4) *Degree of control of environmental conditions*											
**Environment-**change	127	31.8	11.3	88	39	18.2	10.2	4.9	3.5	9.2	6.2
**Environment-**unchanged	478	27.3	10.3	180	296	12.8	8.7	4.1	2.4	6.6	5.9
(5) *Referee influence*											
**Referee-**intervenes directly	281	27.7	10.9	132	155	13.8	9.7	4.0	2.7	7.4	5.8
**Referee-**does not intervene directly	324	28.7	10.5	136	180	14.0	9.0	4.4	2.3	6.7	6.2

*Note: N* = individual numbers, individual = athletes in individual sports, individual-with opp. = athletes in individual sports with a direct opponent, individual-without opp. = athletes in individual sports without a direct opponent, team = athletes in team sports, contact = athletes in contact sports, non-Contact = athletes in non-contact sports, environment-changed = athletes in sports where environment may change in competition, environment-unchanged = athletes in sports with standardized environment in competition, referee intervenes directly = referee intervenes directly in the competition, referee-does not intervene directly = referee does not intervene directly in the competition, *M* = Mean, *SD =* standard deviation, F_(N)_ = number female, M_(N) =_ number male

**Table 2:** Overview of the athletes that participated in the study by sports

Sport	*N*	%	Sport	*N*	%	Sport	*N*	%
Athletics	13	2.1	Fencing	2	0.3	Ropeskipping	1	0.2
Acrobatics	1	0.2	Fitness	10	1.7	Running	10	1.7
Artistic cycling	15	2.5	Floorball	2	0.3	Sailing	3	0.5
Badminton	6	1.0	Football	1	0.2	Shooting sports	1	0.2
Basketball	30	5.0	Field hockey	1	0.2	Skiing	2	0.3
Beach volleyball	21	3.5	Golf	35	5.8	Soccer	45	7.4
Boule	1	0.2	Gymnastics	6	1.0	Softball	1	0.2
Boxing	7	1.2	Handball	99	16.4	Squash	1	0.2
Canoe	1	0.2	Inline skating	4	0.7	Swimming	6	1.0
Cheerleeding	107	17.7	Judo	5	0.8	Taekwondo	2	0.3
Climbing	2	0.3	Jujutsu	5	0.8	Tabletennis	21	3.5
Combat sports	2	0.3	Karate	5	0.8	Tennis	4	0.7
Croquet	1	0.2	Paddle	1	0.2	Traithlon	7	1.2
Dance sports	18	3.0	Parkour	1	0.2	Volleyball	12	2.0
Darts	3	0.5	Racing bike	7	1.2	Water polo	7	1.2
Equitation	64	10.6	Rugby	1	0.2	Weight training	4	0.7

*Note: N =* number of athletes, % = number of athletes in percent

**Table 3:** Descriptive statistics on EI-levels

	*N*	Well-being		Self-control		Emotionality		Sociability		Trait EI	
		*M*	*SD*	*M*	*SD*	*M*	*SD*	*M*	*SD*	*M*	*SD*
**Total**	605	5.63	0.86	4.87	0.85	5.12	0.76	5.01	0.77	5.16	0.61
*Female*	268	5.58	0.89	4.82	0.84	5.19	0.78	4.93	0.78	5.14	0.58
*Male*	335	5.67	0.82	4.91	0.87	5.06	0.76	5.01	0.79	5.18	0.59
(1) **Social interaction**											
**Individual**	272	5.71	0.89	4.93	0.83	5.13	0.78	4.93	0.80	5.17	0.61
*Female*	159	5.61	0.95	4.78	0.78	5.24	0.77	4.86	0.76	5.13	0.64
*Male*	113	5.85	0.78	5.13	0.85	4.97	0.76	5.03	0.84	5.25	0.55
**Team**	333	5.56	0.82	4.82	0.87	5.10	0.75	5.01	0.77	5.15	0.58
*Female*	109	5.55	0.79	4.87	0.92	5.11	0.79	5.03	0.78	5.16	0.56
*Male*	222	5.58	0.84	4.80	0.87	5.10	0.75	5.00	0.76	5.14	0.58
(2) **Presence of an opponent**											
**Individual-** with opp.	69	5.68	0.95	5.12	0.88	5.06	0.81	4.81	0.79	5.19	0.57
*Female*	24	5.63	1.18	4.84	1.08	5.27	0.83	4.78	0.75	5.18	0.72
*Male*	45	5.72	0.82	5.27	0.73	4.95	0.78	4.83	0.81	5.20	0.49
**Individual-** without opp.	208	5.69	0.88	4.87	0.81	5.13	0.77	4.98	0.81	5.17	0.61
*Female*	132	5.58	0.92	4.77	0.73	5.25	0.77	4.85	0.78	5.12	0.62
*Male*	76	5.90	0.77	5.03	0.92	4.94	0.74	5.19	0.84	5.23	0.59
**Team**	328	5.58	0.83	4.82	0.87	5.11	0.75	5.01	0.76	5.15	0.58
*Female*	112	5.58	0.79	4.86	0.91	5.11	0.78	5.06	0.78	5.17	0.58
*Male*	214	5.58	0.82	4.79	0.85	5.12	0.74	4.98	0.75	5.14	0.59
(3) **Physical contact**											
**Contact**	217	5.65	0.83	4.94	0.93	5.07	0.80	4.96	0.83	5.17	0.61
*Female*	101	5.51	0.92	4.87	1.02	5.08	0.83	5.00	0.82	5.13	0.65
*Male*	114	5.80	0.70	4.86	0.87	5.06	0.79	4.93	0.84	5.21	0.57
**Non-contact**	388	5.62	0.87	4.83	0.81	5.14	0.74	4.98	0.76	5.15	0.58
*Female*	167	5.63	0.88	4.79	0.71	5.26	0.74	4.89	0.75	5.15	0.58
*Male*	221	5.60	0.88	4.86	0.87	5.06	0.72	5.05	0.76	5.16	0.58
(4) **Degree of control of environmental conditions**											
**Environment-**change	127	5.58	0.90	4.73	0.76	5.19	0.74	5.02	0.72	5.12	0.58
*Female*	88	5.46	0.95	4.67	0.68	5.25	0.74	4.89	0.70	5.06	0.59
*Male*	39	5.86	0.73	4.86	0.93	5.05	0.72	5.30	0.69	5.25	0.55
**Environment-**unchanged	478	5.64	0.85	4.91	0.87	5.10	0.77	4.96	0.80	5.17	0.59
*Female*	180	5.65	0.86	4.89	0.90	5.16	0.80	4.95	0.81	5.18	0.62
*Male*	296	5.64	0.85	4.92	0.86	5.06	0.75	4.97	0.79	5.17	0.58
(5) **Referee influence**											
**Referee-**intervenes directly	281	5.67	0.81	4.96	0.88	5.08	0.79	4.94	0.80	5.18	0.58
*Female*	126	5.58	0.87	4.87	0.95	5.14	0.81	4.97	0.79	5.16	0.61
*Male*	153	5.76	0.74	5.05	0.81	5.04	0.79	4.90	0.81	5.19	0.54
**Referee-**does not intervene directly	324	5.60	0.89	4.79	0.82	5.14	0.74	5.01	0.76	5.15	0.60
*Female*	142	5.59	0.91	4.77	0.73	5.24	0.75	4.89	0.76	5.13	0.61
*Male*	153	5.60	0.88	4.80	0.89	5.07	0.72	5.10	0.75	5.16	0.60

*Note: N* = individual numbers, individual = athletes in individual sports, individual-with opp. = athletes in individual sports with a direct opponent, individual-without opp. = athletes in individual sports without a direct opponent, team = athletes in team sports, contact = athletes in contact sports, non-Contact = athletes in non-contact sports, environment-changed = athletes in sports where environment may change in competition, environment-unchanged = athletes in sports with standardized environment in competition, referee intervenes directly = referee intervenes directly in the competition, referee-does not intervene directly = referee does not intervene directly in the competition, *M* = Mean, *SD =* standard deviation, 30-item questionnaire utilizing a 7-point Likert scale (1 = completely disagree, 7 = completely agree)

### Summary of statistical analysis

To examine the differences in athletes’ EI levels among various types of sports, a series of five one-way ANOVAs were conducted. The results of these analyses are presented separately for each of the five group constellations.

#### Social interaction

The social interaction was significantly related to the EI dimension well-being, *F*(1, 605) = 4.03, *p* =.05, *η²* = 0.01. Athletes in individual sports (*MW* = 5.71, *SD* = 0.89) had significantly higher scores of well-being compared to athletes in team sports (*MW* = 5.56, *SD* = 0.82). Furthermore, there was a significant main effect of gender on the EI dimension well-being, namely that males (*MW* = 5.67, *SD* = 0.82) had significantly higher levels of well-being than females (*MW* = 5.58, *SD* = 0.89), *F*(2, 605) = 4.25, *p* =.02, *η²* = 0.01. However, there was no significant interaction between gender and *sport type, *F*(1, 605) = 1.85, *p* =.17, suggesting that the relationship between the EI dimension well-being and type of sport was not moderated by gender. There were no significant differences in the levels of the EI dimensions emotionality, sociability, self-control, and the global trait EI scores between individual sports and team sports.

#### Presence of an opponent

There were no significant differences in the levels of the EI dimensions emotionality, sociability, self-control, well-being, and the global trait EI scores regarding the presence of an opponent.

#### Physical contact

The degree of physical contact was significantly related to the EI dimension self-control, *F*(1, 605) = 3.94, *p* =.05, *η²* = 0.00. Athletes in contact sports (*MW* = 4.94, *SD* = 0.93) had significantly higher levels for self-control than athletes in non-physical contact sports (*MW* = 4.83, *SD* = 0.81). Furthermore, there was a significant main effect of age. The older the athletes were, the higher their level of self-control, *F*(1, 605) = 6.21, *p* =.01, *η²* = 0.01. The degree of physical contact did not significantly relate to the EI dimensions emotionality, sociability, well-being, and the global trait EI scores.

#### Degree of control of environmental conditions

The degree of control of environmental conditions significantly related to the EI dimension self-control, *F*(1, 605) = 3.73, *p* =.05, *η²* = 0.01. Athletes in sports with standardized conditions in competition (*MW* = 4.91, *SD* = 0.87) had significantly higher levels for self-control than athletes in sports where environment conditions can change in competition (*MW* = 4.73, *SD* = 0.76). Furthermore, there was a significant main effect of age. The older the athletes were, the higher their level of self-control, *F*(1, 605) = 6.81, *p* =.01, *η²* = 0.01. The degree of control of environmental conditions did not significantly relate to the EI dimension emotionality, sociability, well-being, and the global trait EI scores.

#### Referee influence

The degree of referee influence significantly related to the EI dimension self-control, *F*(1, 605) = 6.87, *p* =.01, *η²* = 0.01. Athletes in sports where a referee intervenes directly in the competition (*MW* = 4.96, *SD* = 0.88) had significantly higher scores for self-control than athletes in sports without direct referee intervention (*MW* = 4.79, *SD* = 0.82). Furthermore, there was a significant main effect of age. The older the athletes were, the higher their level of self-control, *F*(1, 605) = 5.16, *p* =.02, *η²* = 0.01. No significant differences were found in the levels of EI dimensions, including emotionality, sociability, well-being, and the global trait EI scores, between sports with direct referee intervention in the competition and sports without direct referee intervention. The main effects of all one-way ANOVA analyses are presented in Table 4.

**Table 4:** Results of One-way ANOVA

	Well-being				Self-control				Emotionality				Sociability				Trait EI		
*df*	*F*	*p*	η²		*F*	*p*	η²		*F*	*p*	η²		*F*	*p*	η²		*F*	*p*	η²
**Social interaction**: Individual (*N* = 272) vs. Team (*N* = 333)																			
1	4.03	0.05	0.01		1.44	0.23	0.00		0.03	0.86	0.00		0.84	0.36	0.00		1.14	0.71	0.00
**Presence of an opponent**: Individual- with opp. (*N* = 69) vs. Individual- without opp. (*N* = 208) vs. Team (*N* = 328)																			
2	1.94	0.15	0.01		1.96	0.14	0.01		0.05	0.95	0.00		2.11	0.12	0.01		2.12	0.81	0.00
**Physical contact**: Contact (*N* = 217) vs. Non-contact (*N* = 388)																			
1	0.55	0.46	0.00		3.94	0.05	0.01		1.70	0.19	0.00		0.41	0.84	0.00		0.31	0.58	0.00
**Degree of control of environmental conditions**: Environment-change (*N* = 127) vs. Environment-unchanged (*N* = 478)																			
2	0.01	0.91	0.00		3.73	0.05	0.01		1.94	0.66	0.00		3.20	0.07	0.01		0.29	0.59	0.00
**Referee influence**: Referee-intervenes directly (*N* = 281) vs. Referee-does not intervene directly (*N* = 324)																			
1	1.27	0.26	0.00		6.87	0.01	0.01		1.03	0.31	0.00		0.84	0.36	0.00		0.56	0.46	0.00

*Notes: df =* degrees of freedom, *F =* F-value, *p =* *p*-value, *η²* = Eta squared, *N* = individual numbers, individual = athletes in individual sports, individual-with opp. = athletes in individual sports with a direct opponent, individual-without opp. = athletes in individual sports without a direct opponent, team = athletes in team sports, contact = athletes in contact sports, non-Contact = athletes in non-contact sports, environment-changed = athletes in sports where environment may change in competition, environment-unchanged = athletes in sports with standardized environment in competition, referee intervenes directly = referee intervenes directly in the competition, referee-does not intervene directly = referee does not intervene directly in the competition, 30-item questionnaire utilizing a 7-point Likert scale (1 = completely disagree, 7 = completely agree). The mean values and standard deviations for each EI dimension across the different sport classifications are reported in Table 3

## Discussion

The primary objective of this study was to investigate the differences in athletes’ EI levels among five key aspects of sports: [1] social interaction [2], presence of an opponent [3], physical contact [4], degree of control over environmental conditions, and [5] referee influence. By employing appraisal theories of emotions, the study aimed to identify and categorize sports groups based on the specific emotional demands associated with each sport. This approach allowed for a comprehensive analysis of the relevance of EI within specific sport types and determined whether certain dimensions of EI exerted particular influence in those sports. The dataset comprised responses from 605 active athletes involved in 147 different sports, ensuring a diverse sample.

Contrary to our initial hypotheses, the results of our analyses did not support significant differences in trait EI scores across different sports, which is consistent with previous studies comparing individual and team athletes [40, 49–51, 65]. The findings indicate that trait EI may have comparable significance across diverse sports [51, 52]. However, it is plausible to posit that the total score of trait EI might lack the specificity to capture the nuanced emotional challenges inherent in different sports [9]. As a result, it is likely that the four dimensions of trait EI hold varying degrees of importance within each sport, thus attenuating the discriminative capacity of the overall trait EI score.

The objective of our study was to examine potential differences in the four dimensions of EI - sociability, emotionality, self-control, and well-being - between athletes participating in individual and team sports. In contrast to the findings of Laborde et al. [51], our results indicated that athletes in individual sports exhibited higher levels of well-being compared to those in team sports. Our findings also contradicted the results reported by Castro-Sánchez et al. [30] and Castro-Sanchez et al. [47], who also identified differences but reported opposing findings. Our study revealed that athletes in individual sports demonstrated higher levels of self-esteem, increased optimism, and greater feelings of happiness, aligning with the findings of Laborde et al. [65], who reported higher levels of self-efficacy, self-esteem, positivity, resilience, and perseverance among athletes in individual sports. A potential explanation for these findings is the varying degrees of freedom experienced by athletes in their daily routines. Team athletes generally have less control over the structure of training and competition, particularly concerning recovery and workload. Team sports tend to provide less consideration for individual mental and emotional stress. In contrast, individual athletes have greater influence over the intensity and volume of their training, allowing them to incorporate their exertion levels into the planning process. This higher degree of freedom in individual sports may contribute positively to athletes’ overall well-being [90].

Moreover, the coach-athlete relationship may also contribute to these findings. Individual athletes often develop a close and intense bond with their coach, which can directly enhance their well-being. In contrast, team sports typically involve less frequent and brief individual interactions with the coach, while individual athletes receive ongoing personalized feedback. This increased level of specificity in feedback can positively influence the well-being of individual athletes [90]. Additionally, team athletes frequently encounter competitive situations and internal conflicts within the team, which can have a direct negative impact on both their performance and training, potentially influencing their well-being adversely [61].

Our findings indicated that athletes in contact sports, sports with standardized competition conditions, and sports with direct referee intervention demonstrated significantly higher levels of self-control compared to athletes in other sports. This result is consistent with previous research suggesting that athletes in team sports with contact exhibit superior emotional regulation skills compared to those in non-contact sports [30]. Studies focusing on EI in combat sports have also supported this observation [45, 86, 91, 92].

The higher levels of self-control observed in athletes engaged in contact sports can be explained by several factors. Contact sports involve direct physical confrontations with opponents, necessitating a controlled response to physical attacks. Impulsivity and emotional outbursts can result in harm to both players and should be avoided [45, 54, 93]. Additionally, athletes must regulate their emotions and cope with stress to adhere to their strategic plans and avoid falling into destructive emotional cycles [9, 94]. Finally, athletes must regulate any anxiety stemming from prior experiences, injuries, or acute physical pain to maintain focus and intensity during competition [93, 95].

Contrary to our expectations, our findings regarding sports with standardized competition conditions diverge from the assumption that better self-control skills are required to respond to changing environmental conditions that can complicate performance and pose health risks [78, 79]. This is in contrast to the results of Marczak and Ginszt [96], which reported higher effectiveness in coping with stress and emotions during climbing competitions. However, the dynamic nature of changing environmental conditions may contribute to athletes perceiving a lack of full control, leading to a more critical evaluation of their self-control in competition.

Our findings further indicate that athletes participating in sports with direct referee influence demonstrate significantly higher levels of self-control in comparison to athletes in sports without direct referee influence. While research in sports psychology has primarily focused on the experiences and stress management strategies of referees [97], we can only speculate that this result may stem from the need to effectively manage referee decisions, which can induce stress [83] and impact the athlete’s affective state [9].

### Limitations

Several limitations should be acknowledged in the present study. Firstly, the cross-sectional design precludes the establishment of causal relationships. Future research using longitudinal or experimental designs would provide stronger evidence for causal interpretations and enhance understanding of the directionality of potential associations. For example, it could investigate whether training and competition participation in specific sports promote the development of dimensions of EI, how this process unfolds over time, and whether the effect is less pronounced in other sports or follows a different developmental trajectory. Secondly, the relatively low response rate of the online survey (19.1%) raises concerns about the generalizability of the findings. However, it should be noted that low response rates in online surveys do not necessarily imply low representativeness [98]. Nonetheless, efforts should be made in future studies to improve response rates and enhance participant diversity. A further criticism is that the sample comprised exclusively German participants, which limits the generalizability of the findings. Therefore, future studies should also engage with this issue and investigate the extent to which cultural differences may influence the current results or lead to alternative conclusions. Lastly, the categorization of sports relied on theoretical assumptions derived from appraisal theories of emotions rather than empirical research. While these theoretical frameworks offer valuable insights, further investigation should focus on validating the theoretically derived categories for different sport groups to confirm or, if needed, modify them. Studies incorporating objective measures and empirical evidence would strengthen the validity and robustness of the sport categorization process.

### Implications

The present study indicates that considering the emotional demands across different sports when comparing EI levels can be an effective approach, as it incorporates the context in which the effects of EI are expected to manifest. The findings further suggest that the overall EI score offers limited explanatory value, while individual dimensions may reveal significant differences between sports groups. Notably, the study highlights the significance of the self-control dimension of EI in sports, suggesting its prominent role compared to other dimensions. Future research should prioritize investigating self-control when studying the impact of EI in sports, systematically incorporating potential mediators such as established self-control strategies (e.g., mindfulness practices, mental training techniques, and coping strategies) [99, 100].

Replication of this study is recommended to gain deeper insights into the psychological competencies required in specific sports. In this context, it is essential to focus on identifying the mechanisms through which high levels of the EI dimensions of self-control confer advantages to athletes who possess them. Additionally, it is crucial to examine the processes through which the EI dimension of self-control influences specific emotions, athletes’ emotional experiences, or emotional dynamics. This can be achieved, for example, through process models that incorporate potential mediators (e.g., specific coping strategies) or moderators (e.g., competition experience).

It is important for future studies to analyze the emotional demands experienced in different sports, including pre-, during, and post-competition, as well as during training, using appraisal theories of emotions as a framework [101]. These studies should also consider the specificities related to age and gender to accurately identify the emotional demands of each sport and determine key factors for optimal athletic performance. We also assume that identifying emotion-specific performance parameters for individual sports may be beneficial in this context, as it would facilitate more precise investigations into which dimensions of EI are most pertinent to performance in each sport. Building on this, it seems worthwhile to conduct a more detailed analysis of the specific manifestations and mechanisms of EI in individual sports, particularly with regard to the role of self-control and potential mediators and moderators that may influence its effects. Ultimately, these findings can contribute to developing categorizations for comparing sport types based on EI and other relevant psychological factors. Additionally, longitudinal studies are needed to explore the developmental trajectory of EI and its dimensions within the sporting context.

## Conclusion

The present study aimed to explore the differences in athletes’ EI levels among various types of sports using a cross-sectional sample. Drawing on the appraisal theory of emotions, the study identified sport-specific emotional demands and developed a theoretical framework for categorizing sports based on these demands. The findings revealed that athletes in contact sports, standardized competitions, and sports with direct referee involvement had higher scores in the self-control dimension of EI. Athletes in individual sports showed higher scores in the well-being dimension of EI. However, no significant differences were found in the total score of EI or the dimensions of emotionality and sociability. These results highlight the importance of self-control in competitive sports with respect to EI. The implications of these findings emphasize the need to understand the unique emotional demands of different sports and promote collaboration among coaches, athletes, sport managers, and applied sport psychologists to enhance the effectiveness of sports practice.

## Electronic supplementary material

Below is the link to the electronic supplementary material.

Supplementary Material 1

## Data Availability

The datasets utilized and/or analyzed in this study are accessible from the corresponding author upon reasonable request.
