# Supplementary material for: Trait emotional intelligence in competitive sports: are there differences in dimensions of emotional intelligence when comparing different sports?
Source: BMC Psychol. 2025 Mar 15;13:253. doi: 10.1186/s40359-025-02563-w (PMC11909853; doi:10.1186/s40359-025-02563-w)
Supplement: Supplementary file 1 — Supplementary Material 1 [file 40359_2025_2563_MOESM1_ESM.docx]

**Appendix A** Factorial and Subscale Structure of the TEIQue by Petrides (2009)

| **Facets** | **High scorers view themselves as . . .** |
| --- | --- |
| **well-being** |  |
| self-esteem | …successful and self-confident |
| trait optimism, | …confident and likely to “look on the bright side” of life |
| trait happiness | …cheerful and satisfied with their lives |
| **self-control** |  |
| emotion regulation | … capable of controlling their emotions |
| stress management | … capable of withstanding pressure and regulating stress |
| impulsiveness (low) | … reflective and less likely to give in to their urges |
| **emotionality** |  |
| emotion perception (self and others) | … clear about their own and other people’s feelings |
| emotion expression | … capable of communicating their feelings to others |
| trait empathy | … capable of taking someone else’s perspective |
| relationships | … capable of maintaining fulfilling personal relationships |
| **sociability** |  |
| assertiveness | … forthright, frank, and willing to stand up for their rights |
| social awareness | … accomplished networkers with superior social skills |
| emotional management (others) | … capable of influencing other people’s feelings |

*Note.* Adopted from Petrides, 2009, page 14.
